# A Study on the Longitudinal Relationship between Changes in Depression and Cognitive Function among Older Adults Living Alone

**DOI:** 10.3390/healthcare11202712

**Published:** 2023-10-11

**Authors:** Soyoung Park, Kyuhyoung Jeong, Seoyoon Lee

**Affiliations:** 1 Department of Social Welfare, Semyung University, 65 Semyung-ro, Jecheon 27136, Republic of Korea; nillyria@semyung.ac.kr; 2 Interdisciplinary Graduate Program in Social Welfare Policy, Yonsei University, 50 Yonsei-ro, Seodaemun-gu, Seoul 03722, Republic of Korea; sylee92@yonsei.ac.kr

**Keywords:** cognitive function, depression, older adults, Korea

## Abstract

Background: As South Korea faces unprecedented population aging, this longitudinal study examined the relationship between depression and cognitive function changes in older individuals living alone. Methods: The study used data from the Korean Longitudinal Study of Aging (KLoSA). A total of 1354 participants with data available over a period of at least three years, from 2016 (wave 1) to 2020 (wave 8), were included, and latent growth modeling (LGM) was used for analysis. Results: Depression levels increased gradually among older individuals living alone and cognitive function declined over time among older adults living alone. Moreover, higher initial depression levels were associated with lower initial cognitive function levels and a more rapid cognitive decline over time. Therefore, it is imperative that depression be addressed as a potential cause of cognitive impairment and dementia. Furthermore, rapid increases in depression corresponded to rapid declines in cognitive function, indicating the need for continuous monitoring and intervention in cases of escalating depression, as it may negatively affect cognitive abilities. Conclusions: These findings highlight the complex interplay between depression and cognitive function among older individuals living alone. Policy support to encourage participation in these programs is crucial to enhance the well-being of this vulnerable population.

## 1. Introduction

Approximately 10% of the world’s population were estimated to be over 65 by 2022, and this proportion is forecast to increase to 16% by 2050 [1]. Although the aging trend is a global phenomenon, in Korea aging has been accelerating at a rate that has never been seen before. Korean society became an ‘aging society’ in 2000 when the aging population constituted more than 7% of the population. Just 17 years later, it became an ‘aged society’, with the aging population representing over 14%, according to the Organization for Economic Co-operation and Development (OECD) definition [2]. With over 20% of the population expected to be aged 65 or older by 2026, Korea will become a ‘super-aged society’, and thus one of the world’s oldest societies [3]. In addition to the many social concerns associated with the rapid growth of the aging population, there is a decline in cognitive function among older adults. The cognitive dysfunction associated with aging ranges from mild cognitive impairment to dementia, and mild cognitive impairment is highly likely to progress to dementia [4]. While cognitive decline in older adults cannot be equated with dementia, it can be said that dementia is the final stage of cognitive decline. Mild cognitive decline can negatively impact the quality of life of older adults and their families [5,6,7], and when it progresses to dementia, it poses a threat to the quality of life of older adults and their families, especially those family members who provide primary care [8]. Indeed, the World Health Organization estimates that more than 55 million people worldwide suffer from dementia, and dementia-related costs amount to USD 1.3 trillion [9]. Korea is also experiencing rapid aging, which is leading to a sharp increase in cognitive impairment and dementia [10]. Between 2015 and 2019, the percentage of older Koreans diagnosed with dementia rose from 5.9% to 7.3% [11]. According to estimates from 2022, the total number of dementia patients, including those who are not officially diagnosed, will reach over 935,000, accounting for 10.38% of the aging population [12]. By 2050, this figure is expected to exceed 16% [13]. As a result of recognizing the severity of dementia, the Korean government has adopted a policy that provides social care for dementia patients through the implementation of a national dementia responsibility system [14].

Acknowledging these global shifts, several studies have identified depression as a significant contributing factor to cognitive impairment, including dementia [15,16,17,18,19,20]. A decline in economic capabilities and social relationships is common among older adults due to physical aging, illnesses, and stressors such as the loss of a spouse or friends. There has been a gradual decline in the traditional practice of extended families living together in South Korea, resulting in a reduced level of intimacy within the household. Many of these factors can lead to depressive symptoms among older adults, and depression can contribute to cognitive decline, ultimately resulting in mild cognitive impairment and dementia among older adults [21,22,23,24,25]. However, despite this, depression in the aging population is often perceived as a part of the aging process, and there is limited involvement and attention from families and society [26].

In particular, older individuals living alone have been reported to experience higher rates of depression than those living with their families [19,27,28]. Those who live alone face loneliness as a result of the lack of social support and protection from their families, as well as depression as a result of the challenges of living alone, especially if their health is poor or if they have underlying health conditions [29]. The cognitive function of older people who live alone is also more likely to decline [30]. Indeed, recent research indicates that social isolation, notably social isolation with little interaction with people or social activities, has a more detrimental effect on cognitive function than living alone itself [31,32]. Another study on social isolation suggested that living alone can easily result in social isolation and that may have a detrimental effect on cognitive function [33]. In the case of South Korea, research indicates that older individuals living alone suffer from a greater degree of depression than older individuals living with their children or older couples [34]. There has been a steady increase in the percentage of older people living alone in South Korea, reaching 34.9% in 2022, and this percentage is projected to continue to rise in the future [35]. Therefore, depression experienced by older individuals living alone is likely to worsen, further exacerbating the relationship between depression and cognitive decline.

In order to effectively intervene in the field of depression and cognitive function among older adults, there is a need to understand how depression levels and cognitive function change in older adults and how these changes are interrelated in a longitudinal manner. There have been a number of studies exploring depression and cognitive function in older adults; however, there is limited research into how depression and cognitive function change with aging, especially in the context of South Korean older individuals. Research on the evolution of depression over time has often presented conflicting results, with some studies indicating that depression levels increase as old age advances [36,37,38,39], whereas others suggest that depression does not change with age [40]. Steenland and colleagues (2012) [23] suggested that recent depressive episodes within the past two years play an important role in the progression from normal cognitive function to mild cognitive impairment or dementia. Rather than solely focusing on a history of depression, they emphasized the importance of addressing current depression.

Considering the limited longitudinal studies of depression and cognitive function changes in Korean older individuals, particularly in the vulnerable subgroup of older individuals living alone, research on depression and changes in cognitive function in older people living alone is urgently needed. Thus, this study aims to longitudinally examine the relationship between changes in depression and changes in cognitive function among a group of older people living alone in Korea. On the basis of the results of this study, we discuss social intervention measures that can be used to slow the increase in depression and prevent cognitive decline among senior citizens living alone.

This study is based on the following hypotheses:

1. How do depression and cognitive function change over time?
2. Is there a relationship between the initial value and rate of change in depression and the initial value and rate of change in cognitive function?

## 2. Materials and Methods

### 2.1. Data

Using data from the Korean Longitudinal Study of Aging (KLoSA), which covered waves 1 through 8, this study examined the relationship between depression and cognitive function in older individuals living on their own. A representative panel survey of older adults in South Korea, the Korean Longitudinal Study of Aging (KLoSA) is intended to provide data necessary for effective socioeconomic policy formulation by quantifying and understanding the many aspects of older adults’ lives, including their socioeconomic, psychological, demographic, and health-related characteristics. KLoSA has conducted a survey every two years during the months of August and December. The target population for KLoSA is people aged 45 or older who live throughout the country. Using a laptop, computer-assisted personal interviews (CAPIs) were conducted, and stratified cluster sampling was used to collect samples. This study focused on individuals living alone aged 65 and older as of the first wave in 2006. This study included a final sample of 1354 participants with data available for assessing depression changes and cognitive function changes over a period of at least three years, from 2016 (wave 1) to 2020 (wave 8). The data on major variables were excluded from the analysis when there were missing values.

The demographic characteristics of the study participants are based on data from the year 2006 and are summarized in Table 1. In terms of gender, 580 individuals (42.8%) were male and 774 individuals (57.2%) were female, indicating that there were more females than males in the sample. For education level, the majority of the participants, specifically 1011 individuals (74.7%), had completed education up to elementary school or below. More participants resided in urban areas, i.e., 875 individuals (64.6%), compared to 479 individuals (35.4%) living in rural areas. The average age of the participants was 73.50 years, with a standard deviation of 5.78 years. The average annual household income was KRW 684.84 million, with a standard deviation of KRW 1360.88 million.

### 2.2. Variables

#### 2.2.1. Independent Variable: Depression

A depression score of CES-D10 (The Center for Epidemiological Studies-Depression Scale) was used to assess depression in the study. In the Korean Longitudinal Study of Aging (KLoSA), depression is measured using the Korean version of the CES-D-10, which consists of 10 shortened items. The CES-D was developed by Radloff (1977) [41]. The 20 questions of the CES-D were translated into Korean and used as the CES-D 10 Boston version by Kohout et al. (1993) [42]. There is a four-point rating system for depression: 1 indicates that it occurs rarely or never, 2 means that it occurs sometimes or a little, 3 indicates that it occurs occasionally or a moderate amount of the time, and 4 means that it occurs most of the time. The higher the score on the depression scale, the greater the degree of depression. For each year in this study, the reliability of the depression scale was as follows: 2006 (0.857), 2008 (0.875), 2010 (0.878), 2012 (0.874), 2014 (0.854), 2016 (0.856), 2018 (0.868), and 2020 (0.831).

#### 2.2.2. Dependent Variable: Cognitive Functions

As the dependent variable in this study, cognitive function was assessed using the K-MMSE (Korean Mini-Mental State Examination), which is an adaptation of the MMSE (Mini-Mental State Examination) originally developed by Folstein, Folstein, and McHugh in 1975 [43]. A Korean version of the K-MMSE was developed by Kang, Na, and Hahn in 1997 [44]. This 30-point questionnaire is widely used in clinical and research settings to assess cognitive function [45]. A higher score on the K-MMSE indicates a better level of cognitive function. K-MMSE is the same as MMSE, with six categories (time/place orientation, memory registration (short-term memory), attention and calculation ability, memory recall (long-term memory), language (reading, writing, speaking), and drawing). A K-MMSE total score of 24 to 30 indicates no cognitive impairment, a score of 18 to 23 indicates mild cognitive impairment, and a score of 0 to 17 indicates severe cognitive impairment [46].

### 2.3. Statistical Analysis

Data were analyzed using the Stata 15.1 and M-plus 8.0 programs for this study, and the analysis methods and procedures were as follows: To explore the demographic characteristics of the study population and the characteristics of key variables, descriptive statistics were initially derived. Secondly, latent growth modeling (LGM) was used to estimate depression and cognitive function changes over time and to examine the longitudinal relationship between depression and cognitive function. A number of fit indices were used to evaluate the goodness of fit of the latent growth model, including the Tucker–Lewis Index (TLI), the Comparative Fit Index (CFI), and the Root Mean Square Error of Approximation (RMSEA).

## 3. Results

### 3.1. Descriptive Statistics

The descriptive statistical analysis of depression revealed that the average depression score was lowest in 2020, at 1.82 (SD = 0.51), and highest in 2008, at 2.00 (SD = 0.63). The average depression scores fluctuated between 2006 and 2020, showing periods of both increase and decrease (Table 2). Cognitive function scores were highest in 2006 at 22.30 (SD = 6.14), while lowest in 2020 at 19.32 (SD = 6.43). Between 2006 and 2020, the average cognitive function scores consistently decreased, indicating that cognitive function has been declining.

### 3.2. Analysis of Research Model

In this study, latent growth modeling was conducted in two steps. To estimate the initial depression values and rate of change in cognitive function for older individuals living alone, an unconditional model analysis was performed first. Based on the initial values and rates of change obtained in the first step, a conditional model analysis was conducted to examine the relationship between changes in depression and changes in cognitive function.

#### 3.2.1. Unconditional Model Analysis

Before conducting conditional model analysis, an unconditional model analysis was performed to understand the changes in depression and cognitive function among older individuals living alone. A linear growth model and a no-growth model were analyzed in order to identify the most appropriate growth pattern. The results showed that the linear growth models provided a better fit for both depression (χ^2^ = 186.860, *p* < 0.001, CFI = 0.911, TLI = 0.920, RMSEA = 0.061) and cognitive function (χ^2^ = 170.782, *p* < 0.05, CFI = 0.958, TLI = 0.962, RMSEA = 0.058) compared to the no-growth models. Accordingly, linear growth models were adopted in order to better explain changes in depression and cognitive function over time (Table 3).

Based on the selected linear growth models, the following results were observed regarding patterns of change and individual differences in depression and cognitive function. For depression, the average initial depression score for older individuals living alone was 1.938 (*p* < 0.001), with a variance of 0.198 (*p* < 0.001) (Table 4). These findings were statistically significant, indicating significant differences in the initial depression scores among the study participants. The average rate of change in depression for older individuals living alone was 0.011 (*p* < 0.01), with a variance of 0.007 (*p* < 0.001). This implies that over time, depression tends to increase among older individuals, but the rate of change varies among study participants. The covariance between the initial depression score and the rate of change was −0.007 (*p* < 0.001), indicating that older individuals with higher initial depression scores tend to experience gradual increases in depression over time. As a result, individuals with lower initial depression scores experienced a greater increase in depression over time.

For cognitive function, the average initial cognitive function score for older individuals living alone was 22.399 (*p* < 0.001), with a variance of 26.435 (*p* < 0.001). There was a difference in the initial cognitive function scores among study participants, which was statistically significant. It was found that the average rate of change in cognitive function for older individuals living alone was −0.746 (*p* < 0.001), with a variance of 0.954 (*p* < 0.001). This suggests that, over time, cognitive function tends to decrease among older individuals, but the rate of change varies by individuals. There was no significant covariance between the initial cognitive function score and the rate of change.

#### 3.2.2. Conditional Model Analysis

In the conditional model analysis, the impact of the initial values and rate of change in depression on the initial values and rate of change in cognitive function among older individuals living alone was examined. In the results of the conditional model fit analysis, RMSEA = 0.050, CFI = 0.925, TLI = 0.927, and ISAS = 0.925, indicating that the model fit the data well (Table 5).

The initial values of depression were found to have a significant impact on both the initial values and rate of change in cognitive function (Coef. = −6.136, *p* < 0.001 for initial values; Coef. = −0.648, *p* < 0.001 for rate of change). In other words, lower initial depression values were associated with higher initial cognitive function values, and cognitive function gradually declined over time. Conversely, higher initial depression values were associated with lower initial cognitive function values, and cognitive function decreased more rapidly over time.

Furthermore, the rate of change in depression was found to be significantly related to the rate of change in cognitive function (Coef. = −7.976, *p* < 0.001). Cognitive function also decreased gradually over time as depression increased gradually. The decline in cognitive function was more pronounced and rapid when depression increased rapidly over time.

## 4. Discussion

This study aimed to longitudinally examine the relationship between depression and cognitive function changes among older individuals living alone. The significance of this study lies in its longitudinal examination of depression and cognitive function changes in Korean older individuals, particularly focusing on those living alone, who are vulnerable to depression and dementia.

Depression increased over time among older individuals living alone according to the study. This finding aligns with previous research on depression in individuals living alone [19,27,28,34], as well as the literature on depression changes in older adults [36,37,38,39]. The results of these studies highlight the need for social attention and intervention for depression among older individuals living alone. This group should be provided with continuous depression prevention programs. This study also found that individuals with lower initial depression scores experienced a rapid increase in depression over time. The results of this study are indicative of the characteristics and vulnerabilities of older individuals living alone. When dealing with various challenges on their own, they often experience loneliness since they lack the support and protection of family members [28,29]. Despite their initial depression levels being low, the significant increase in depression was due to the stress and difficulties associated with living alone. The results of this study suggest that depression prevention policies should not exclude older individuals who live alone, even if their depression levels are initially assessed as low. Therefore, it is suggested that all older individuals living alone, regardless of their initial level of depression, should undergo regular depression assessments and programs to reduce depression.

Secondly, the results of the study indicated that cognitive function declined over time among older adults living alone. This is consistent with previous research on cognitive function in older adults [23]. Due to the prolonged period of living alone and the natural process of aging, older adults living alone may experience accelerated cognitive decline. It is highly relevant to the context of older individuals living alone that the study of Bennett et al. (2002) [4] suggests that older adults with mild cognitive impairment have a threefold increased risk of developing dementia. Accordingly, it is essential to consider the significant implications of this study for older adults living alone, especially in light of the increased risk of dementia as a result of living alone. Many programs are being implemented in senior welfare centers, health centers, dementia care centers, and nursing homes in South Korea to preserve cognitive function and prevent dementia [47]. It is, however, still difficult to find programs and policies tailored specifically to the needs of aging individuals living alone who suffer from cognitive impairment. It is imperative that programs to maintain cognitive function in aging individuals living alone are developed and implemented in light of the difficulties of daily life caused by cognitive impairment, the strain on families, and the societal costs associated with dementia [8,9]. 

Thirdly, the study’s examination of the longitudinal relationship between depression and cognitive function revealed that higher initial depression levels were associated with lower initial cognitive function levels and a more rapid decline in cognitive function over time. These findings are consistent with previous research indicating that depression is a significant factor influencing cognitive impairment and dementia [15,16,17,18,19,20]. These findings are also applicable to older individuals living alone in South Korea. These findings emphasize the need for more proactive social interventions since depression in older individuals living alone can have long-term and short-term cognitive effects. Therefore, it is necessary to establish a system that regularly assesses the level of depression among older individuals through senior welfare centers, health centers, dementia care centers. Moreover, programs for depression treatment and cognitive enhancement should be provided to those with high levels of depression along with the assessment.

Fourth, as depression increased rapidly over time among older individuals living alone, cognitive function also decreased rapidly. These research findings are supported by previous studies on depression and cognitive function among older adults [21,22,23,24,25]. Further, these results are consistent with research conducted by Chen and colleagues (2008) [36], which showed that although mild depression symptoms do not necessarily cause dementia, severe depression can have a significant impact on it. Therefore, depression should be monitored continuously, especially if it increases rapidly over time, since it may negatively impact cognitive abilities. As part of the National Health Insurance Corporation’s health check-up program, steps should be taken to ensure that older individuals who live alone are not overlooked. Support for monitoring depression changes should also be considered as part of policy development. Moreover, there is a need to conduct substantial research on the factors that affect depression in older adults and to develop intervention strategies based on the results of such research. Depression in older individuals living alone can be reduced by implementing practical interventions, such as balanced diets, exercise, mood-enhancing training, and communication training [48]. There is a need for tailored health promotion programs, mobility support to prevent social isolation, regular home visits by social workers or volunteers, and programs that facilitate the development of social relationships. Encouraging the participation of older individuals in these programs requires policy support.

This study distinguishes itself from previous studies by examining 16 years of depression changes over time, as well as the relationship between depression and cognitive function. In addition, this study emphasizes the importance of interventions for older individuals living alone and provides directions for possible interventions based on the research findings. There are, however, limitations to this study, as it was unable to control for changes in depression and cognitive function of seniors living alone due to the COVID-19 outbreak and the external effects of the pandemic during the period of analysis from 2006 to 2020. Additionally, there was a high sample attrition rate over a period of 16 years. Future studies should address these limitations by conducting follow-up studies.

## 5. Conclusions

The purpose of this study was to longitudinally examine the relationship between changes in depression and changes in cognitive function in older people living alone, who are susceptible to depression and cognitive decline, over the course of 16 years. The first finding was that depression in older individuals living alone increased over time, particularly in those with initially low levels of depression, for whom depression escalated rapidly over time. This indicates that there is a need for continuous depression management and intervention for all older individuals living alone, regardless of their initial depression levels. Secondly, cognitive function in older individuals living alone decreased over time. Taking into consideration the age group of older adults, tailored programs should be developed and implemented for the preservation of cognitive function and the prevention of dementia. Furthermore, it has been found that as initial depression levels increase in older individuals living alone, cognitive function decreases rapidly. On the basis of these research results, a system for regular depression assessments and proactive management of vulnerable older individuals with high levels of depression is recommended. Finally, it has been observed that cognitive function declines rapidly over time as depression increases rapidly among older individuals living alone. The implementation of practical measures to prevent and manage depression would assist in preserving cognitive function. It is also recommended that policy support be provided to encourage older adults living alone to participate in these programs.

## Figures and Tables

**Table 1:** Demographic characteristics of study participants. (N = 1354).

	Categories	N	%
Gender	Male	580	42.8
	Female	774	57.2
Education Level	Elementary School or below	1011	74.7
	Middle School	131	9.7
	High School	136	10.0
	University Graduates or above	76	5.6
Residential Area	Urban	875	64.6
	Rural	479	35.4
Age (M (SD))		73.50 (5.78)	
KRW Annual Household Income (M (SD))		684.84 (1360.88)	

**Table 2:** Descriptive analysis of major variables.

Year	Depression			Cognitive Function		
	N	Mean	SD	N	Mean	SD
2006	1354	1.86	0.61	1354	22.30	6.14
2008	1325	2.00	0.63	1296	21.62	6.27
2010	1120	1.99	0.63	1066	21.16	6.65
2012	950	1.98	0.61	901	21.16	6.69
2014	765	1.92	0.59	707	20.17	7.07
2016	588	1.85	0.58	540	20.47	6.91
2018	382	1.90	0.60	356	19.47	7.26
2020	178	1.82	0.51	172	19.32	6.43

**Table 3:** Goodness of fit of latent growth models of cognitive function.

Model		χ^2^	df	CFI	TLI	RMSEA
Depression	No-Growth Model	349.262 ***	34	0.821	0.852	0.083
	Linear Growth Model	186.860 ***	31	0.911	0.920	0.061
Cognitive Function	No-Growth Model	837.723 ***	34	0.756	0.799	0.132
	Linear Growth Model	170.782 *	31	0.958	0.962	0.058

* *p* < 0.05, *** *p* < 0.001.

**Table 4:** Mean and variance of the initial score and rate of change in unconditional models.

Variables		Mean		Variance		Covariance
		Estimate	S.E.	Estimate	S.E.	
Depression	Initial Score	1.938 ***	0.015	0.198 ***	0.012	−0.007 **
	Rate of Change	0.011 **	0.004	0.007 ***	0.001	
Cognitive Function	Initial Score	22.399 ***	0.158	26.435 ***	1.331	0.322
	Rate of Change	−0.746 ***	0.044	0.954 ***	0.097	

** *p* < 0.01, *** *p* < 0.001.

**Table 5:** Path coefficient of study model.

			Coef.	S.E.
Depression initial value	→	Cognitive function initial value	−6.136 ***	0.409
Depression initial value	→	Cognitive function Rate of change	−0.648 ***	0.121
Depression rate of change	→	Cognitive function Rate of change	−7.976 ***	0.702

*** *p* < 0.001.

## Data Availability

The datasets generated during and/or analyzed in this study are publicly available upon request from: https://www.koweps.re.kr:442/eng/data/data/list.do (accessed on 10 May 2023).
